# Postnatal Nutrition to Improve Brain Development in the Preterm Infant: A Systematic Review From Bench to Bedside

**DOI:** 10.3389/fphys.2019.00961

**Published:** 2019-07-26

**Authors:** Lisa M. Hortensius, Ruurd M. van Elburg, Cora H. Nijboer, Manon J. N. L. Benders, Caroline G. M. de Theije

**Affiliations:** ^1^Department of Neonatology, University Medical Center Utrecht, Utrecht University, Utrecht, Netherlands; ^2^Emma Children's Hospital, Amsterdam UMC, University of Amsterdam, Amsterdam, Netherlands; ^3^Danone Nutricia Research, Utrecht, Netherlands; ^4^Laboratory of Neuroimmunology and Developmental Origins of Disease, University Medical Center Utrecht, Utrecht University, Utrecht, Netherlands

**Keywords:** nutrition, preterm infants, animal models, clinical, pre-clinical, brain injury, neurodevelopment

## Abstract

**Background:** Preterm infants are at high risk for Encephalopathy of Prematurity and successive adverse neurodevelopmental outcome. Adequate nutrition is crucial for healthy brain development. Maternal breast milk is first choice of post-natal enteral nutrition for preterm infants. However, breast milk contains insufficient nutrient quantities to meet the greater nutritional needs of preterm infants, meaning that supplementation is recommended.

**Aim:** To provide an overview of current literature on potential nutritional interventions for improvement of neurodevelopmental outcome in preterm infants, by taking a bench to bedside approach from pre-clinical models of neonatal brain injury to randomized controlled clinical trials (RCTs) in preterm infants.

**Methods:** Separate clinical and pre-clinical searches were performed in Medline and Embase for English written papers published between 08/2008 and 08/2018 that studied a single nutritional component. Papers were included if one of the following components was studied: lipids, carbohydrates, proteins, vitamins, minerals, probiotics, prebiotics, oligosaccharides, fatty acids, or amino acids, with brain injury, brain development or neurodevelopmental outcome as outcome measure in preterm infants (gestational age <32 weeks and/or birth weight <1,500 g) or in animal models of neonatal brain injury.

**Results:** In total, 2,671 pre-clinical studies and 852 RCTs were screened, of which 24 pre-clinical and 22 RCTs were included in this review. In these trials supplementation with amino acids and protein, lipids, probiotics (only clinical), prebiotics (only clinical), vitamins, and minerals was studied. All included pre-clinical studies show positive effect of supplementation on brain injury and/or neurodevelopment. Although some nutrients, such as glutamine, show promising short term outcome in clinical studies, no evident long term effect of any supplemented nutrient was found. Main limitations were inclusion of studies no older than 10 years at time of search and studies that focused on single nutritional components only.

**Conclusion:** Even though many pre-clinical trials demonstrate promising effects of different nutritional interventions on reducing brain injury and/or improving neurodevelopmental outcome, these positive effects have so far not evidently been demonstrated in RCTs. More clinically relevant animal models and long term follow up after clinical trials are needed to move novel nutritional therapies from bench to bedside of preterm infants.

## Introduction

Preterm birth is commonly defined as any birth before 37 completed weeks of gestation and has been estimated to account for 10% of all births (Liu et al., 2012). Over the past decade, advances in neonatal care have led to decreased mortality of preterm infants in Western society (Euro-Peristat Project with SCPE EUROCAT, 2013). Therefore, the main focus of neonatal care has changed from reducing mortality to reducing long-term morbidity. Brain injury and successive impaired neurodevelopmental outcome is a major morbidity in the preterm infant (Twilhaar et al., 2018c). Despite improvements in preventative strategies (e.g., prenatal steroids) and enhanced neuromonitoring, the rate of neurodevelopmental impairments remains high and is estimated to be 25% in extremely preterm infants born before 28 weeks of gestation (Glass et al., 2015).

During the third trimester of gestation, the brain undergoes a rapid trajectory of growth. Brain growth from 30 to 40 weeks of gestation is greatest in the cerebellum, which increases 258% in size (Kersbergen et al., 2016). The total brain volume is increased by 140% during these 10 weeks. Third trimester brain volumes have been shown to be reduced in preterm infants compared to healthy control fetuses, and growth trajectories were slower in cerebrum, cerebellum, brain stem and intracranial cavity (Bouyssi-Kobar et al., 2016). Injury to the preterm brain is commonly defined as “Encephalopathy of Prematurity” (EoP) and includes different pathologies. The most commonly observed pathology is cerebral white matter injury (WMI), which can present as diffuse or as punctate, cystic, or hemorrhagic lesions. In addition, also germinal matrix-intraventricular hemorrhages, and cerebellar disturbances are frequently observed in the preterm brain (van Tilborg et al., 2016). The pre- and post-natal conditions leading to EoP are typically associated with cerebral oxygen fluctuations and systemic inflammation (van Tilborg et al., 2016). These risk factors are thought to lead to an arrest in oligodendrocyte maturation, followed by a myelination failure of neuronal axons (van Tilborg et al., 2018b), resulting in reduced neuronal connectivity and brain volume. Both neuronal connectivity and brain volume are important predictors of neurodevelopmental outcome in the preterm infant.

As the rapidly growing brain at this stage in particular is vulnerable for nutrient insufficiencies (Georgieff, 2007), one may hypothesize that nutrient intake by preterm infants may predict brain growth and possibly neurodevelopmental outcomes. Intake quantities of selective and total nutrients during early life of preterm infants were shown to be positively associated with increased head circumference growth (Dabydeen et al., 2008), brain growth and basal nuclei volumes (Coviello et al., 2018; Schneider et al., 2018). In addition, nutrient intake was also positively correlated with higher fractional anisotropy (FA) values in selected white matter tracts (Schneider et al., 2018), such as the posterior limb of internal capsule (PLIC) (Coviello et al., 2018), and with greater axonal diameters in the corticospinal tract, indicating improved white matter integrity (Dabydeen et al., 2008). Moreover, cumulative protein intake was positively associated with higher cognitive and motor scores (Coviello et al., 2018). In a different study, it was demonstrated that first week protein and energy intakes were associated with improved developmental outcomes in extremely preterm born infants (Stephens et al., 2009). Together, these studies highlight the importance of adequate nutrition and the balance between protein, fat, and caloric content for early life brain development of the preterm infant.

Currently, “adequate” nutrition for the preterm infant is designed to “provide nutrients to approximate the rate of growth and composition of weight gain for a normal fetus of the same post-menstrual age and to maintain normal concentrations of blood and tissue nutrients” (American Academy of Pediatrics, 2009). The preferred source of nutrition for preterm infants is human milk from the infant's own mother. However, it has been long known that very/extremely preterm infants fed exclusively breast milk cannot match intrauterine growth patterns and may end up with extra uterine growth restriction. Therefore, human milk is fortified with a Human Milk Fortifier (HMF) to enhance protein, caloric, vitamin and mineral intake. It is important to note that extremely preterm infants are fed fortified human milk, regardless of the actual content of the human milk itself. From a Cochrane systematic review on the use of HMF for preterm nutrition it was concluded that HMF slightly increased in-hospital growth rates, but there was not enough evidence to suggest that feeding preterm infants with a standard amount of multi-nutrient fortified breast milk improves important developmental outcomes (Brown et al., 2016). This highlights the knowledge gap between what we currently feed the preterm infant and what has actually been proven to be effective on developmental outcome. The importance of this issue has been acknowledged by the World Health Organization (WHO). In their recommendations on interventions to improve preterm birth outcomes (World Health Organization, 2015), they pose the following two questions as research priorities: (1) “What are the optimal feeding methods for preterm infants with birth weight <1,200 g?” and (2) “Is there a role for total parenteral nutrition in the management of preterm infants?”

In recent years, there has been increased interest in how to optimally feed the preterm infant, especially with respect to neurodevelopment. Randomized controlled trials (RCTs) have been focused on increasing protein, caloric, or fat intake and on supplementation with specific nutritional components. Also in pre-clinical studies, the potential mechanisms in which various specific nutritional supplements can protect or treat the neonatal brain from injury have been investigated in recent years. Therefore, this systematic review aimed to provide an overview of the current literature on potential nutritional interventions for the improvement of neurodevelopmental outcome in preterm infants, by taking a bench to bedside approach from pre-clinical models of neonatal brain injury to randomized clinical trials in preterm infants.

## Methods

### Search

This systematic review was conducted according to PRISMA guidelines (Moher et al., 2009). To evaluate the clinical impact of nutritional interventions on brain development and neurodevelopmental outcome, Embase and Medline (Pubmed) were searched on August 15 2018 for RCTs published in the last 10 years. The search was divided into a pre-clinical search and a clinical search. For the pre-clinical search, studies were included if they met the following inclusion criteria: (1) neonatal brain injury induced by hypoxia, ischemia, inflammation and/or hyperoxia, induced during gestation or in the neonatal period (no later than post-natal day 10 (P10), which corresponds to near term human neurodevelopment) (Salmaso et al., 2014), (2) post-natal intervention with a nutritional component that started between birth and weaning (P21) with one of the following nutritional components: lipids, carbohydrates, proteins, vitamins, minerals, probiotics, prebiotics, oligosaccharides, fatty acids or amino acids, and (3) brain injury or neurodevelopment as outcome measure. Studies were excluded if (1) the article was written in a language other than English (2) the article was published over 10 years ago at time of search or if (3) more than one nutritional component was studied as the aim of this review was to study individual nutritional components. Embase and Medline were both searched with similar search strategies. For example, for Medline the following search was used:

(((((“Brain Injuries”[Mesh]) OR ((((“Brain”[Mesh]) OR brain[Title/Abstract])) AND ((damage[Title/Abstract]) OR injury[Title/Abstract])))) AND ((“animal experimentation”[MeSH Terms] OR “models, animal”[MeSH Terms] OR “invertebrates”[MeSH Terms] OR “Animals”[Mesh:noexp] OR “animal population groups”[MeSH Terms] OR “Mice”[Mesh] OR “Rats”[Mesh] OR “Rodentia”[Mesh] OR mice[Title/Abstract] OR mouse[Title/Abstract] OR rodent[Title/Abstract] OR rodents[Title/Abstract] OR rat[Title/Abstract] OR rats[Title/Abstract] OR murine[tiab] OR “Papio”[Mesh] OR baboon[Title/Abstract] OR baboons[Title/Abstract] OR marmoset[tiab] OR marmosets[tiab] OR monkey[tiab] OR monkeys[tiab] OR primate[Title/Abstract] OR primates[Title/Abstract] OR “Primates”[Mesh] OR “Sheep, Domestic”[Mesh] OR sheep[Title/Abstract] OR lamb[Title/Abstract] OR lambs[Title/Abstract] OR pigs[tiab] OR pig[tiab] OR swine[tiab] OR swines[tiab] OR piglets[tiab] OR piglet[tiab] OR boar[tiab] OR boars[tiab] OR “guinea pigs”[tiab] OR “guinea pig”[tiab] OR rabbits[tiab] OR rabbit[tiab]))) AND (((“Infant, Low Birth Weight”[Mesh] OR “small for gestational age”[tiab] OR “small for date”[tiab] OR sga[tiab] OR “low birthweight”[tiab] OR vlbw[tiab] OR elbw[tiab] OR “Infant, Newborn”[Mesh] OR “Intensive Care, Neonatal”[Mesh] OR “Intensive Care Units, Neonatal”[Mesh] OR “Neonatal Nursing”[Mesh] OR infant^*^[tiab] OR newborn^*^[tiab] OR neonat^*^[tiab] OR prematur^*^[tiab] OR preterm^*^[tiab] OR “Infant, Premature”[Mesh] OR “Infant, Very Low Birth Weight”[Mesh] OR “low birth weight”[tiab]))))) AND (((((((((((((((((((((((“Lipids”[Mesh]) OR lipid[Title/Abstract]) OR lipids[Title/Abstract])) OR (((“Carbohydrates”[Mesh]) OR carbohydrate[Title/Abstract]) OR carbohydrates[Title/Abstract])) OR (((“Proteins”[Mesh:NoExp]) OR protein[Title/Abstract]) OR proteins[Title/Abstract])) OR (((“Vitamins”[Mesh]) OR vitamin[Title/Abstract]) OR vitamins[Title/Abstract])) OR ((((“Probiotics”[Mesh]) OR beneficial bacteria[Title/Abstract]) OR probiotics[Title/Abstract]) OR probiotic[Title/Abstract])) OR (((“Prebiotics”[Mesh]) OR prebiotic[Title/Abstract]) OR prebiotics[Title/Abstract])) OR (((“Minerals”[Mesh]) OR mineral[Title/Abstract]) OR minerals[Title/Abstract])) OR (((“Oligosaccharides”[Mesh]) OR oligosaccharide[Title/Abstract]) OR oligosaccharides[Title/Abstract])) OR (((“Amino Acids”[Mesh]) OR amino acid[Title/Abstract]) OR amino acids[Title/Abstract])) OR ((fatty acid[Title/Abstract]) OR fatty acids[Title/Abstract])) OR ((“Diet”[Mesh]) OR diet^*^[Title/Abstract])) OR ((“Enteral Nutrition”[Mesh]) OR enteral[Title/Abstract])) OR ((“Parenteral Nutrition”[Mesh:NoExp]) OR parenteral[Title/Abstract])) OR (((“Micronutrients”[Mesh]) OR micronutrient[Title/Abstract]) OR micronutrients[Title/Abstract])) OR ((nutrient[Title/Abstract]) OR nutrients[Title/Abstract])) OR ((macronutrient[Title/Abstract]) OR macronutrients[Title/Abstract])) OR energy[Title/Abstract]) OR fortif^*^[Title/Abstract]) OR nutrition^*^[Title/Abstract])).

For the clinical search, inclusion criteria were as follows: (1) preterm infants born before 32 weeks of gestation and/or birth weight below 1,500 g, (2) a post-natal nutritional intervention that was started between day of birth and 28 days post-natal and that contained one of the following nutritional components: lipids, carbohydrates, proteins, vitamins, minerals, probiotics, prebiotics, oligosaccharides, fatty acids, or amino acids, and (3) brain development, brain injury or neurodevelopmental outcome as outcome measure. As with the pre-clinical search, exclusion criteria were: (1) non-English written papers, (2) papers older than 10 years at time of the search, and (3) studies that researched more than one nutritional component. Embase and Medline were both searched with similar search strategies. For example, for Medline the following search was used:

((((((“Infant, Low Birth Weight”[Mesh] OR “small for gestational age”[tiab] OR “small for date”[tiab] OR sga[tiab] OR “low birthweight”[tiab] OR vlbw[tiab] OR elbw[tiab] OR prematur^*^[tiab] OR preterm^*^[tiab] OR “Infant, Premature”[Mesh] OR “Infant, Very Low Birth Weight”[Mesh] OR “low birth weight”[tiab])))) AND (((((((((((((((((((((((((“Lipids”[Mesh]) OR lipid[Title/Abstract]) OR lipids[Title/Abstract])) OR (((“Carbohydrates”[Mesh]) OR carbohydrate[Title/Abstract]) OR carbohydrates[Title/Abstract])) OR (((“Proteins”[Mesh:NoExp]) OR protein[Title/Abstract]) OR proteins[Title/Abstract])) OR (((“Vitamins”[Mesh]) OR vitamin[Title/Abstract]) OR vitamins[Title/Abstract])) OR ((((“Probiotics”[Mesh]) OR beneficial bacteria[Title/Abstract]) OR probiotics[Title/Abstract]) OR probiotic[Title/Abstract])) OR (((“Prebiotics”[Mesh]) OR prebiotic[Title/Abstract]) OR prebiotics[Title/Abstract])) OR (((“Minerals”[Mesh]) OR mineral[Title/Abstract]) OR minerals[Title/Abstract])) OR (((“Oligosaccharides”[Mesh]) OR oligosaccharide[Title/Abstract]) OR oligosaccharides[Title/Abstract])) OR (((“Amino Acids”[Mesh]) OR amino acid[Title/Abstract]) OR amino acids[Title/Abstract])) OR ((fatty acid[Title/Abstract]) OR fatty acids[Title/Abstract])) OR ((“Diet”[Mesh]) OR diet^*^[Title/Abstract])) OR ((“Enteral Nutrition”[Mesh]) OR enteral[Title/Abstract])) OR ((“Parenteral Nutrition”[Mesh:NoExp]) OR parenteral[Title/Abstract])) OR (((“Micronutrients”[Mesh]) OR micronutrient[Title/Abstract]) OR micronutrients[Title/Abstract])) OR ((nutrient[Title/Abstract]) OR nutrients[Title/Abstract])) OR ((macronutrient[Title/Abstract]) OR macronutrients[Title/Abstract])) OR energy[Title/Abstract]) OR fortif^*^[Title/Abstract]) OR nutrition^*^[Title/Abstract]))))))) AND ((((“Brain”[Mesh]) OR brain[Title/Abstract])) OR (((“neurodevelopmental outcome”[Title/Abstract]) OR “neurodevelopmental outcomes”[Title/Abstract]) OR neurodevelopment[Title/Abstract]))) AND (((randomized controlled trial[pt] OR controlled clinical trial[pt] OR randomized[tiab] OR placebo[tiab] OR drug therapy[sh] OR randomly[tiab] OR trial[tiab] OR groups[tiab]))).

### Study Selection

Two authors (LH and CdT) reviewed all titles and abstracts independently. If an inconsistency occurred during abstract or full text analysis, consensus was reached in a meeting or a third author (RvE) was consulted. Quality of the included studies was assessed using the Cochrane Risk of Bias assessment tool (Higgins et al., 2011) and the adapted SYCLE's Risk of Bias tool (Hooijmans et al., 2014), for assessing risk of bias for the clinical and pre-clinical studies, respectively. Graphs for risk of bias for individual studies and across studies were made [Review Manager (RevMan) [computer program], 2014].

### Data Collection and Data Items

Included studies were categorized in the results section based on the nutritional component.

From all pre-clinical papers, the following data were extracted (by CdT): nutritional component and amount used as intervention, duration of intervention, duration of follow up, test used to assess neurodevelopmental outcome and/or method to detect brain injury, type of brain injury (hypoxic, ischemic, inflammatory, and/or hyperoxic) and post-natal day of insult.

For all clinical studies, the following data were extracted (by LH): nutritional component and amount used as intervention, duration of intervention, duration of follow up, test used to assess neurodevelopmental outcome, number of patients, gestational age and birth weight of patients and neurodevelopmental outcome.

### Principal Summary Measures

Our primary outcome measure was brain injury and/or neurodevelopmental outcome. For the pre-clinical search, brain injury was defined as either brain volume loss or myelination impairments. Secondary outcomes on brain injury include cell death, oligodendrocyte numbers, glial activation, and cytokine levels. Neurodevelopmental outcome was included in this review if assessed with a validated behavioral task.

For the clinical search, brain injury was defined as either brain volume loss, or brain injury defined by a brain injury scoring system as reported in the original paper. Neurodevelopmental outcome was included in this review if assessed with a validated neurodevelopmental outcome scale. No statistical analysis was done. Outcome was reported as described by the original authors and no authors were contacted.

## Results

### Study Selection

#### Pre-clinical Trials

**Figure 1:**
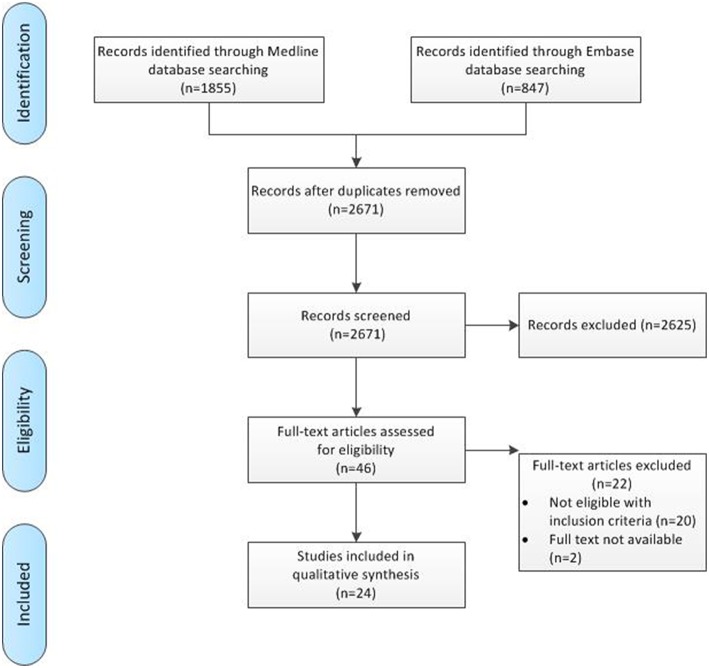
Flowchart of pre-clinical search process.

The pre-clinical search through Medline and Embase provided 1,855 and 847 results, respectively. After screening of title and abstract, 46 papers potentially met our in- and exclusion criteria and were screened as full text to assess eligibility, after which 24 were included in final analysis. Figure 1 gives an overview of the screening process, including reason of exclusion.

#### Clinical Trials

**Figure 2:**
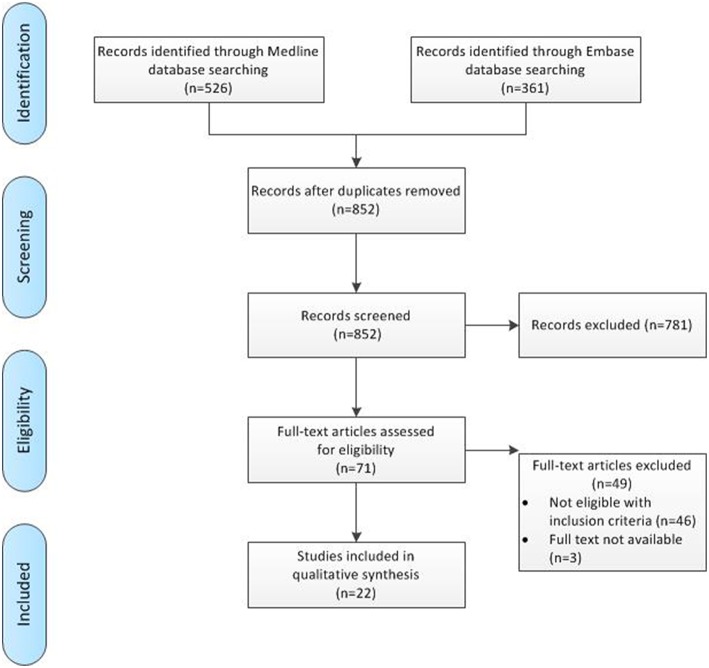
Flowchart of clinical search process.

The clinical search through Medline and Embase provided 526 and 361 results, respectively. After screening of title and abstract, 71 papers potentially met our in- and exclusion criteria and were screened as full text to assess eligibility, after which 22 were included in final analysis. Figure 2 gives an overview of the screening process, including reason of exclusion.

**Figure 3:**
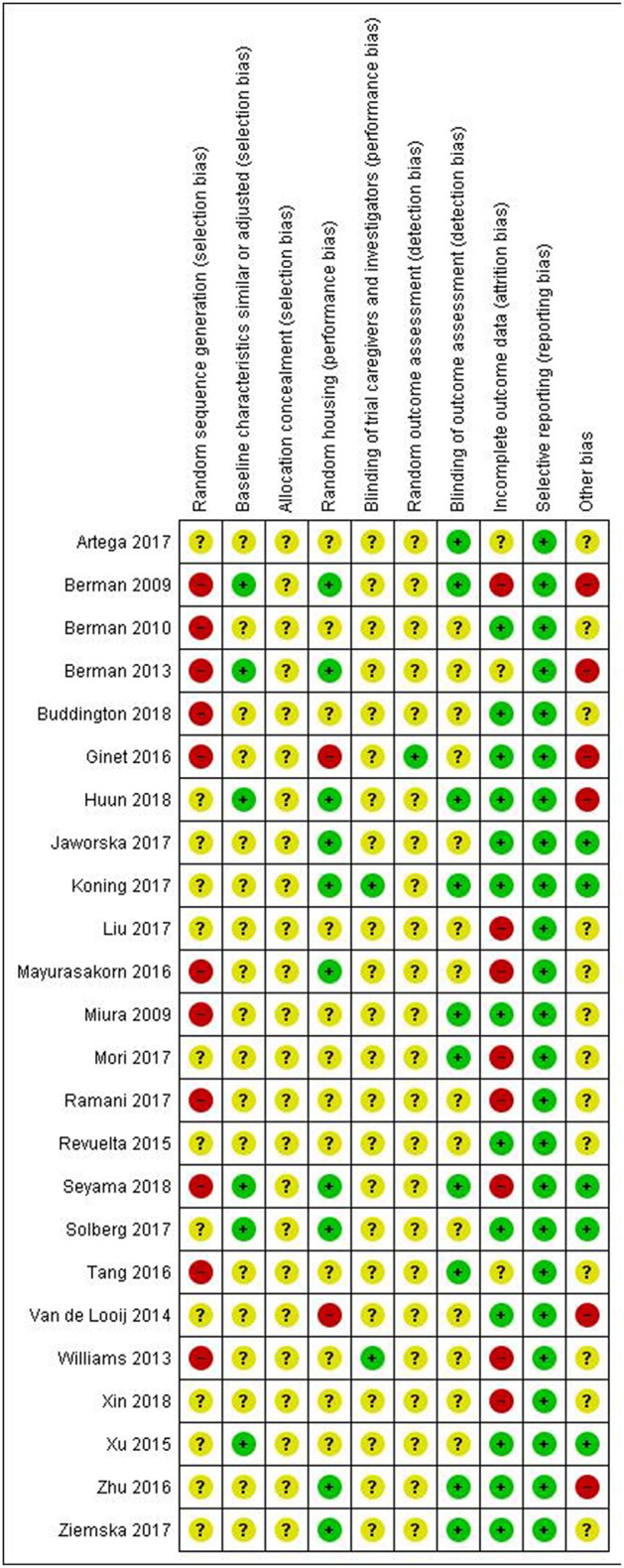
Risk of bias of individual pre-clinical studies.

**Figure 4:**
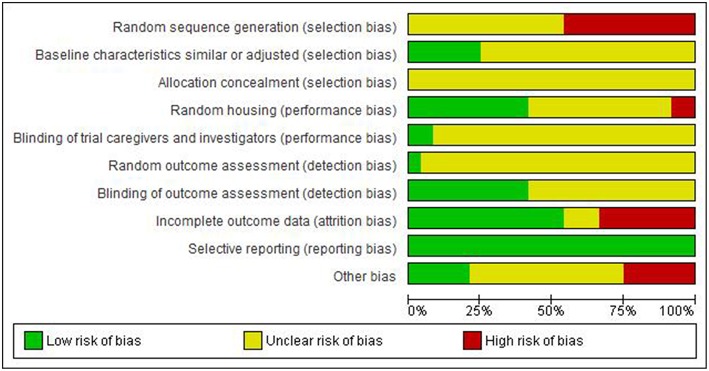
Risk of bias across pre-clinical studies.

**Figure 5:**
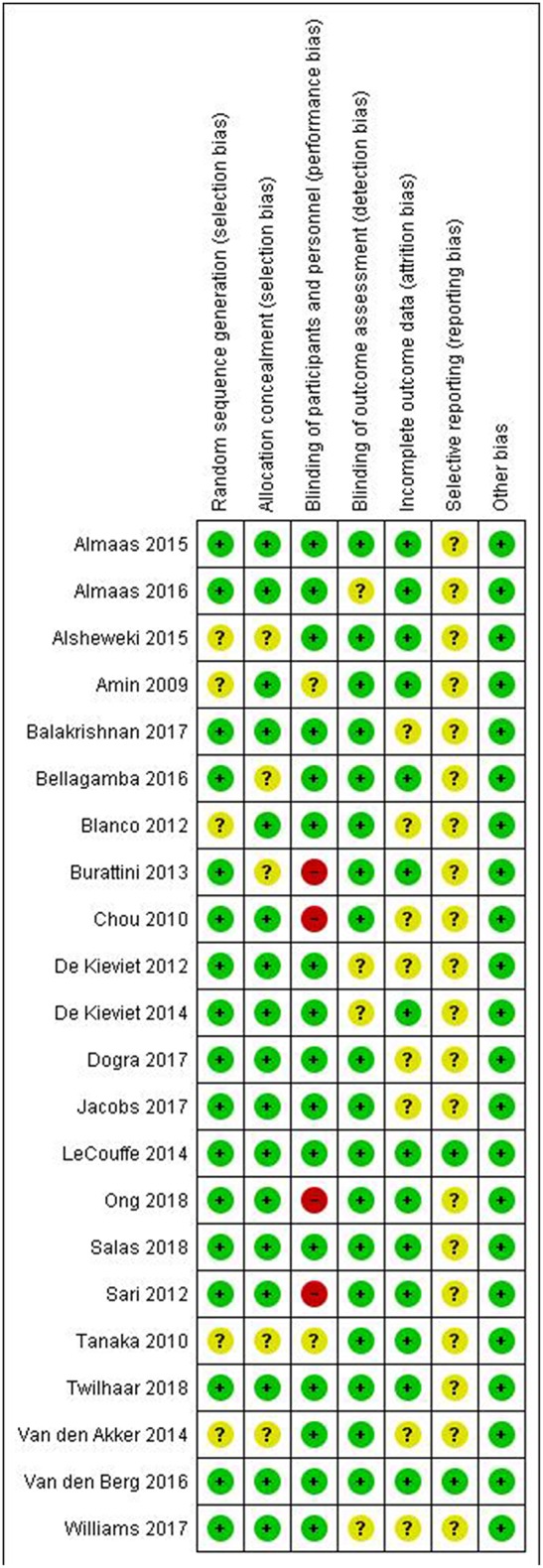
Risk of bias of individual clinical studies.

**Figure 6:**
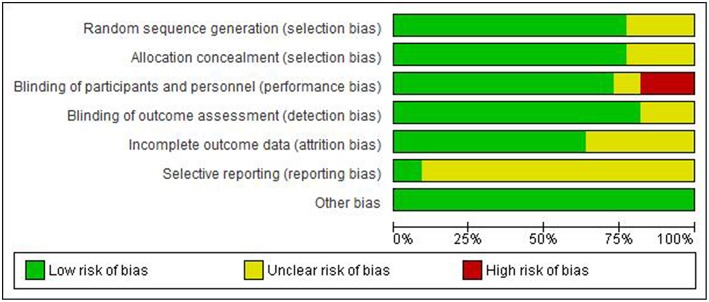
Risk of bias across clinical studies.

Figures 3–6 show the results of the quality assessment for the pre-clinical and clinical studies. No studies were excluded as a result of quality assessment.

### Study Characteristics

#### Pre-clinical Trials

The pre-clinical search resulted in the inclusion of 24 studies. Of those, eight evaluated proteins (including single amino acids), 10 studies evaluated lipids, two studies evaluated probiotic metabolites, two studies evaluated minerals and two studies evaluated vitamins. There were no studies that evaluated supplementation with carbohydrates, probiotics, or prebiotics. The clinical characteristics of the included studies are summarized in Table 1.

**Table 1:** Baseline characteristics of included pre-clinical studies.

**Study**	**Nutritional component**	**Timing (and mode) of administration**	**Brain injury model**	**Primary outcome measure**
van de Looij et al., 2014	Lactoferrin	Pre- and post-treatment (enteral, in maternal diet)	HI P3 rat	Lesion size, myelination
Ginet et al., 2016	Lactoferrin	Pre- and post-treatment (enteral, in maternal diet)	LPS i.c. P3 rat	Lesion size, myelination
Xu et al., 2015	Acetyl-l-carnitine	Post-treatment (s.c.)	HI P7 rat	Lesion size
Tang et al., 2016	Acetyl-l-carnitine	Post-treatment (s.c.)	HI P7 rat	Lesion size, behavior
Zhu et al., 2016	Taurine	Post-treatment (i.p.)	HI P7 rat	Lesion size
Mori et al., 2017	Glycine	Pre-treatment (i.p.)	HI P7 rat	Lesion size
Liu et al., 2017	L-cysteine	Post-treatment (i.p.)	HI P7 mouse	Lesion size, behavior
Xin et al., 2018	L-cysteine	Post-treatment (i.p.)	HI P7 mouse	Lesion size, behavior
Berman et al., 2009	DHA	Pre-treatment (i.p.)	HI P7 rat	Lesion size, behavior
Revuelta et al., 2016	DHA	Pre-treatment (i.p.)	HI P7 rat	Myelination
Arteaga et al., 2017	DHA	Pre-treatment (i.p.)	HI P7 rat	Lesion size, myelination, behavior
Berman et al., 2010	DHA	Pre-treatment (i.p.)	HI+ LPS P7 rat	Lesion size, behavior
Williams et al., 2013	tri-DHA tri-EPA	Post-treatment (i.p.)	HI P10 mouse	Lesion size
Mayurasakorn et al., 2016	tri-DHA tri-EPA	Post-treatment (i.p.)	HI P10 mouse	Lesion size, behavior
Berman et al., 2013	DHA	Post-treatment (i.p.)	HI P7 rat	Lesion size, behavior
Solberg et al., 2017	DHA	Post-treatment (i.v.)	HR newborn piglet	Lipid peroxidation
Huun et al., 2018	DHA	Post-treatment (i.v.)	HR newborn piglet	Lipid peroxidation
Buddington et al., 2018	PS-DHA	Post-treatment (enteral)	Preterm-born piglet	Brain weight, myelination
Jaworska et al., 2017	Na-butyrate	Post-treatment (i.p.)	HI P7 rat	Lesion size
Ziemka-Nalecz et al., 2017	Na-butyrate	Post-treatment (i.p.)	HI P7 rat	Behavior
Ramani et al., 2017	Vitamin A	Post-treatment (enteral)	Hyperoxia P2–14 mouse	Behavior
Miura et al., 2009	Vitamin C	Pre-treatment (i.p.)	HI P7 rat	Macroscopical lesion size
Koning et al., 2017	MgSO_4_	Pre-treatment (i.p.)	HI P7 rat	Lesion size
Seyama et al., 2018	MgSO_4_	Pre-treatment (i.p.)	HI P6 rat	Myelination

*i.p., intraperitoneal; i.v., intravenous; s.c., subcutaneous; i.c., intracranial; HI, hypoxia-ischemia; HR, hypoxia-reoxygenation; P, postnatal day; DHA, docosahexaenoic acid; EPA, eicosapentaenoic acid; tri, triglyceride; PS, phosphatidylserine; Na, sodium; MgSO_4_, magnesiumsulphate*.

#### Clinical Trials

The clinical search resulted in the inclusion of 22 studies. Of those, 10 studies evaluated proteins (including single amino acids), five studies evaluated lipids, three studies evaluated probiotics, two studies evaluated prebiotics, one study evaluated minerals and one study evaluated vitamins. There were no studies that evaluated only carbohydrate intake. The clinical characteristics of the included studies are summarized in Table 2.

**Table 2:** Baseline characteristics of included clinical studies.

**Study**	**Nutritional component**	**Enteral/parenteral**	**Inclusion criteria**	**Selected infants (*n*)**	**Primary study outcome**	**NDO or brain injury outcome measure**
Blanco et al., 2012	Protein	Parenteral	>24 weeks and <1,000 g	32	Reduction of potassium	NDO 0–24 months
Burattini et al., 2013	Protein	Parenteral	<1,250 g	96	Body seize at 36 weeks PMA	NDO 24 months
Balakrishnan et al., 2017	Protein	Parenteral	<32 weeks and <1,250 g	114	NDO 18–24 months	NDO 18–24 months
van den Akker et al., 2014	Protein	Parenteral	<32 weeks and <1,500 g	111	Short term safety and efficacy	NDO 24 months
Bellagamba et al., 2016	Protein	Both	<1,250 g	164	Weight gain from birth−1,800 g	NDO 24 months
Dogra et al., 2017	Protein	Enteral	<32 weeks or <1,500 g	92	Head growth at 40 weeks PMA	NDO 12–18 months
de Kieviet et al., 2012	Glutamine	Enteral	<32 weeks or <1,500 g	65	Feeding tolerance	Brain volumes and white matter integrity at 8 years
de Kieviet et al., 2014	Glutamine	Enteral	<32 weeks or <1,500 g	52	Feeding tolerance	HC growth at 1 year and brain volumes at 8 years
Twilhaar et al., 2018a	Glutamine	Enteral	<32 weeks or <1,500 g	61	Feeding tolerance	NDO 13 years
Amin et al., 2009	L-arginine	Both	≤32 weeks or ≤1,250 g	132	Necrotizing enterocolitis	NDO 36 months
Ong et al., 2018	Lipids	Parenteral	≤29 weeks	30	Cholestasis	NDO 6–24 months
Almaas et al., 2015	DHA, AA	Enteral	<1,500 g	98	NDO 6 months	NDO, brain volumes and brain maturation at 8 years
Almaas et al., 2016	DHA, AA	Enteral	<1,500 g	98	NDO 6 months	Behavior and white matter integrity at 8 years
Alshweki et al., 2015	AA/DHA ratio	Enteral	25–32 weeks or <1,500 g	45	NDO 24 months	NDO 24 months
Tanaka et al., 2013	Sphingomyelin	Enteral	<1,500 g	24	NDO 0–18 months	NDO 0–18 months
Chou et al., 2010	Probiotics	Enteral	<1,500 g	301	Necrotizing enterocolitis	NDO 3 years
Jacobs et al., 2017	Probiotics	Enteral	<32 weeks and <1,500 g	735	Late onset sepsis	NDO 2–5 years
Sari et al., 2012	Probiotics	Enteral	<33 weeks or <1,500 g	174	Necrotizing enterocolitis	NDO 18–22 months
LeCouffe et al., 2014	Prebiotics	Enteral	<32 weeks or <1,500 g	93	Neonatal infections	NDO 0–12 months
van den Berg et al., 2016	Prebiotics	Enteral	<32 weeks or <1,500 g	77	Neonatal infections	NDO 24 months
Salas et al., 2018	Vitamin D	Enteral	23–27 weeks	91	Vitamin D concentration	NDO 22–26 months
Williams et al., 2017	Iodide	Both	<31 weeks	1,259	NDO 24 months	NDO 24 months

*NDO, Neurodevelopmental outcome; PMA, Postmenstrual age; HC, Head circumference; DHA, Docosahexaenoic acid; AA, Arachidonic acid*.

### Protein Intake (Including Individual Amino Acids)

#### Pre-clinical Trials

The effects of protein or amino acid supplementation on neonatal brain injury were described in eight pre-clinical studies. Of those studies, two studies investigated the effects of supplementation with lactoferrin (van de Looij et al., 2014; Ginet et al., 2016), a protein abundantly present in human milk. Furthermore, three studies were focused on amino acid-based biosynthetic products acetyl-l-carnitine (Xu et al., 2015; Tang et al., 2016) and taurine (Zhu et al., 2016) that are also present in human milk. Additionally, three pre-clinical studies focused on supplementation with the single amino acids glycine (Mori et al., 2017) or L-cysteine (Liu et al., 2017; Xin et al., 2018).

Lactoferrin is a glycoprotein produced by exocrine glands and released at high levels in human milk, especially colostrum (Nagasawa et al., 1972). van de Looij et al. (2014) investigated the effects of maternal lactoferrin supplementation in a rat model of preterm hypoxic-ischemic encephalopathy (HIE), induced at post-natal day 3 (P3, equivalent to 24–28 weeks gestational age in humans) that presents with impaired cortical development and white matter integrity (van de Looij et al., 2014). Lactoferrin (1 g/kg/day) was supplemented in the maternal diet from P0 throughout lactation and resulted in increased levels of lactoferrin in the stomach and serum of the offspring. MRI analysis revealed reduced cerebral edema hours after the insult and reduced cortical loss accompanied by improved diffusivity at post-natal day 25 in neonatal rats supplemented with lactoferrin. Also the impairments in white matter structure were partly restored upon lactoferrin supplementation, as indicated by improved diffusivity and FA values in the external capsule but not in the corpus callosum of injured rats supplemented with lactoferrin compared to control diet. The authors hypothesized that neuroprotective effects of lactoferrin may be driven by its anti-inflammatory properties, as indicated by a reduction of brain TNF-α and IL-6 expression, which resulted in reduced apoptotic cell death, as indicated by reduced caspase 3 activation at 1 day after injury in the lactoferrin-supplemented rats compared to controls. In a following study, the same research group repeated the lactoferrin intervention study in an inflammation-induced rat model of preterm brain injury, using injection of LPS in the subcortical white matter at P3 (Ginet et al., 2016). Lactoferrin supplementation during lactation again provided neuroprotection as shown by reduced ventriculomegaly, lesion size, and myelination deficits. Although lactoferrin supplementation did not reduce astrogliosis and pro-inflammatory cytokine expression, it did reduce microglia activation as compared to the control diet in the LPS-injured neonatal rat brain.

The biosynthetic product acetyl-l-carnitine is also a component of human milk produced from the amino acids lysine and methionine. After consumption, it rapidly enters the brain and its metabolite carnitine facilitates long chain fatty acid metabolism to produce energy. One group described in two studies the effects of acetyl-l-carnitine supplementation in a rat model of neonatal HIE induced at P7 (equivalent to near term in humans) (Xu et al., 2015; Tang et al., 2016). Pups were injected subcutaneously with 4 doses of 100 mg/kg acetyl-l-carnitine at 0, 4, 24, and 48 h post-HI. Treatment with acetyl-l-carnitine reduced lesion size and promoted oxidative cerebral energy production and minimized anaerobic glycolysis, if administered early after the HI insult (Xu et al., 2015). In the second study, acetyl-l-carnitine supplementation again reduced lesion size, and also improved simple reflexes and spatial memory but not social play, when compared to HIE rats that received vehicle (Tang et al., 2016).

Although strictly classified as a biosynthetic compound derived from cysteine and methionine, taurine is often regarded as the second most abundant amino acid in human milk (Rassin, 1981). After consumption, taurine accumulates in the brain in high concentrations (Rassin, 1981), where it has various functions, for example as a neurotransmitter and trophic factor (Wu and Prentice, 2010). One pre-clinical trial investigated the effects of taurine supplementation in a rat model of neonatal HIE induced at P7 (Zhu et al., 2016). Taurine was administered intraperitoneally (i.p.) every 12 h for 2 days post-HI at doses of 30–120 mg/kg. Taurine dose-dependently reduced lesion size, cell death, and oxidative stress in brains of neonatal HIE rats, as compared to vehicle.

In summary, supplementation with lactoferrin from the day of birth was neuroprotective in two models of preterm brain injury, as shown by improvement of neurological outcome on MRI, potentially though inhibition of neuroinflammation. In addition, peripheral administration of amino acid-based biosynthetic products acetyl-l-carnitine and taurine after the insult, reduced lesion size in a rat model of neonatal near term HIE.

The amino acid glycine acts as an important inhibitory neurotransmitter in the brain. The effects of glycine treatment were described in one study in a rat model of neonatal HIE induced at P7 (Mori et al., 2017). Intraperitoneal injections with 800 mg/kg glycine prior to the hypoxic insult induced a large but brief increase in glycine concentrations in the cerebrospinal fluid and subsequently reduced lesion size and neuronal cell death in neonatal HIE rats, when compared to vehicle injections. This neuroprotection by glycine was associated with reduced expression of TNF-α levels at 12 and 24 h post-HI and 3 days later, reduced neuroinflammation was measured by a lower numbers of astrocytes and microglia in the lesioned area.

One group reported in two pre-clinical studies the effects of l-cysteine treatment in a mouse model of neonatal HIE induced at P7 (Liu et al., 2017; Xin et al., 2018). L-cysteine is catalyzed in the brain to produce endogenous hydrogen sulfide (H_2_S), which has been suggested to inhibit inflammation, oxidative stress and apoptosis. Neonatal mice were subjected to HI and given daily intraperitoneal injections with 2.5–5.0 mg/kg l-cysteine for 3 consecutive days, starting immediately post-HI. Treatment with l-cysteine attenuated brain edema, lesion size and neuronal cell death after neonatal HIE, when compared to vehicle (Liu et al., 2017; Xin et al., 2018). L-cysteine treatment also improved behavioral deficits, including neurological reflexes and spatial working memory, in mice with neonatal HIE. The neuroprotective effects of l-cysteine on functional and anatomical outcome in neonatal mice with HIE were associated with improved synapse formation and reduced oxidative stress and neuroinflammation, as measured by microglia and astrocyte infiltration. Furthermore, the treatment effect of l-cysteine was reversed when H_2_S formation was inhibited *in vivo*, indicating that the neuroprotective mechanisms of l-cysteine depend on its function as an H_2_S donor.

In summary, single amino acid supplementations with glycine (as a pre-treatment) and l-cysteine (as a post-treatment) in rodent models of neonatal near term HIE reduced brain injury and neuroinflammation, and the latter treatment also improved behavioral outcome.

#### Clinical Trials

Ten randomized controlled clinical trials (RCTs) described outcome after amino acid or protein supplementation (Amin et al., 2009; Blanco et al., 2012; de Kieviet et al., 2012, 2014; Burattini et al., 2013; van den Akker et al., 2014; Bellagamba et al., 2016; Balakrishnan et al., 2017; Dogra et al., 2017; Twilhaar et al., 2018a). In six trials different *quantities* of amino acids or protein were evaluated, but all trials had different target values for intervention or control group, different starting time of intervention and/or different length of intervention. Three studies (Blanco et al., 2012; Burattini et al., 2013; Balakrishnan et al., 2017) evaluated increased *parenteral* supplementation with amino acids, starting shortly after birth and targeting 4 g/kg/day within the first days of life, compared to control, aiming on 2, 2.5, or 3 g/kg/day within the first days of life. In total, these studies evaluated 242 infants around 18–24 months corrected age. No beneficial effect of high dose of *parenteral* amino acid supplementation on neurodevelopment was found. Burattini et al. (2013) found no difference in neurodevelopmental outcome or growth between the two groups (*n* = 96 in total). Balakrishnan et al. (2017) found no difference in neurodevelopmental outcome (*n* = 114 in total), but did find lower mean weight, length and head circumference at discharge in infants that received high amino acid supplementation. Blanco et al. (2012) found that infants in the intervention group had significantly lower Mental Developmental Index (Bayley Scales of Infant and Toddler Development, second edition) at 18 months corrected age. At 24 months follow up, this difference was no longer present and no other differences in neurodevelopmental outcome between the two groups were observed (*n* = 32 in total). However, during the 24 months follow up period, infants in the intervention group showed significantly lower head circumference and overall growth. One study evaluated a different target level of amino acids. Bellagamba et al. (2016) evaluated enriched (3.5 g/kg/day parenteral and 4.6 g/kg/day enteral) vs. regular (2.5 g/kg/day parenteral and 3.6 g/kg/day enteral) supplementation from day of birth until a weight of 1,800 g was achieved. The intervention included both *parenteral* and *enteral* supplementation. At 2 years corrected age, no difference in neurodevelopmental outcome was seen (*n* = 164 in total). One study assessed the effect of early amino acid supplementation during the first 3 days of life. van den Akker et al. (2014) started *parenteral* supplementation (2.4 g/kg/day) within 2 h after birth in comparison to controls, who started 24–48 h after birth (1.2 g/kg/day, increased to 2.4 g/kg/day on day 3). At 2 years of age, no difference in outcome between both groups was seen (*n* = 111 in total). However, subgroup analysis revealed higher odds of normal outcome for boys treated with early amino acids (OR 6.17 [1.01–38.46]). In contrast, girls in the intervention group had a significantly lower Mental Developmental Index compared to girls in the control group. The paper by Dogra et al. (2017) was the only study evaluating exclusive *enteral* protein supplementation. Infant feedings were supplemented with regular (0.4 g/100 ml) or protein enriched (1.0 g/100 ml) HMF, which started at an enteral feeding volume of 100 ml/kg/day and ended at discharge. At term equivalent age, infants that received protein enriched HMF had significantly greater head circumferences. However, during the 2 year follow up period, no difference in growth nor mental or motor outcome was seen (*n* = 92 in total).

In summary, all studies combined investigated 609 infants and conflicting evidence was found. Most studies found no improvement of neurodevelopmental outcome after early and/or enriched (par)enteral amino acid supplementation or found an adverse effect on growth and head circumference. The single study that described exclusive enteral protein supplementation showed a beneficial effect on head circumference, but not on neurodevelopmental outcome.

Supplementation with a *single* amino acid was described in four papers. de Kieviet et al. (2012, 2014) and Twilhaar et al. (2018a) described in three papers long term follow up after the same RCT (GEEF study), investigating glutamine supplementation (de Kieviet et al., 2012, 2014; Twilhaar et al., 2018a). Infants received enteral glutamine supplementation (0.3 g/kg/day) or placebo between post-natal day 3 and day 30. The effect of glutamine on head circumference growth during the first year of life (*n* = 65 in total) (de Kieviet et al., 2014) and brain volumes at 8 years of age (*n* = 52 in total) (de Kieviet et al., 2012) was evaluated. Glutamine supplementation was associated with increased head circumference growth during the first year of life as well as with increased white matter, hippocampal and brain stem volumes at 8 years. Neurodevelopmental follow up at 13 years of age (*n* = 61 in total) showed comparable outcomes on all domains between glutamine and placebo groups, except for the visuospatial working memory forward span and the parent rated attention skills, which favored the control group (Twilhaar et al., 2018a).

In a different study, Amin et al. (2009) evaluated the effect of 261 mg/kg/day L-arginine compared to placebo during the first 28 days of life on neurodevelopmental outcome at 36 months corrected age (*n* = 132 in total). No significant difference was found in neurodevelopmental outcome between both groups.

In summary, enteral glutamine supplementation showed increased head circumference growth during the first year of life and increased brain volumes at 8 years. However, at 13 years of age, no positive effect of glutamine was found on neurodevelopmental outcome. Working memory and attention skills differed between the glutamine and control group, favoring the control group, but were within the normal range for both groups.

No advantage from supplementation with L-arginine was detected.

### Lipid Intake (Including Fatty Acids)

#### Pre-clinical Trials

The effects of lipid supplementation on pre-clinical neonatal brain injury have been reported in 10 studies, all focused on docosahexaenoic acid (DHA) supplementation only (Berman et al., 2009, 2010, 2013; Williams et al., 2013; Mayurasakorn et al., 2016; Revuelta et al., 2016; Arteaga et al., 2017; Solberg et al., 2017; Buddington et al., 2018; Huun et al., 2018).

In a rat model for neonatal HIE induced at P7, an i.p. injection of 1–5 mg/kg DHA 4 h prior to the insult improved sensorimotor outcome and reduced lesion size (Berman et al., 2009). In a similar neonatal HIE model, 1 mg/kg DHA was administered i.p. at 10 min prior the HI insult, and prevented impairments in the auditory brain stem response and in myelination of the inferior colliculus, a principal midbrain nucleus of the auditory pathway (Revuelta et al., 2016). Neuron morphology and astrogliosis was also measured, but not statistically analyzed. In addition, the group reported in a similar experimental design that DHA pre-treatment reduced lesion size and mitochondrial cell damage at the hippocampal level, and rescued deficits in myelination in the external capsule and striatum and in spatial memory (Arteaga et al., 2017). In a rat model for neonatal inflammatory HIE using LPS injection prior to HI, pre-treatment with DHA improved sensorimotor outcome, but did not reduce lesion size (Berman et al., 2010).

In addition to DHA administration *prior* to the insult, also treatment *after* the insult has been investigated in rodent models of neonatal HIE. Williams et al. (2013) compared post-treatment with DHA or eicosapentaenoic acid (EPA) triglycerides in a mouse model for neonatal HIE induced at P10 (equivalent to near term in humans) (Williams et al., 2013). Triglyceride emulsions allow for slower release of n-3 fatty acids and therefore avoid high levels of n-3 fatty acids that may lead to brain and liver toxicity (Singh et al., 1989; Oliveira et al., 1997). Intraperitoneal treatment with 375 mg/kg of tri-DHA, but not tri-EPA, immediately after the HI insult reduced lesion size. Furthermore, a treatment delay for up to 2 h after the insult provided neuroprotection on histology level, but a further delay failed to reduce lesion size. In a subsequent study, the group demonstrated that neuroprotection by tri-DHA after neonatal HIE was associated with increased DHA content in the mitochondria and DHA-derived bioactive metabolites in the brain (Mayurasakorn et al., 2016). Tri-DHA treatment immediately post-HI reduced oxidative damage in the brain and improved long-term neurological outcomes. Also in combination with hypothermia, DHA supplementation reduced lesion size and improved sensorimotor outcome when compared to treatment with hypothermia only (Berman et al., 2013).

To improve clinical translation of DHA therapy for neonatal brain injury, Solberg et al. (2017) investigated the effects of DHA in a newborn piglet model of severe hypoxia-reoxygenation (Solberg et al., 2017). DHA was administered i.v. at 5 mg/kg in newborn piglets at 4 h after subjection to hypoxia. Lipid peroxidation markers were significantly lower in the hippocampus and cortex of DHA-treated newborn piglets compared to the untreated control group measured at 9.5 h after treatment. These results indicate that DHA has anti-oxidative effects in the brain. Additionally, the group recently reported in a similar experimental design changes in oxidative stress in different brain regions in response to DHA in combination with hypothermia (Huun et al., 2018). In the white matter, regardless of hypothermia treatment, DHA reduced the levels of di-homo-isoprostanes, a product of adrenic acid oxidation. In the cortex, DHA significantly reduced levels of neurofuranes and di-homo-isoprostanes, only when hypothermia was not applied. In contrast to the first study of Solberg et al. (2017), no differences were observed in the hippocampus.

In addition, one study reported the effects of oral DHA supplementation in preterm-born piglets at 92% gestation (considered relevant to 32 week-old preterm infant) (Buddington et al., 2018). Preterm pigs were born by sterile caesarian section and provided with parenteral nutrition for 16–18 h. Thereafter, the source of nutrition was entirely milk replacer. Milk replacer was supplemented with phosphotidylserine (PS)–DHA to provide 58 mg/kg/day, or sunflower oil as the placebo. Based on rat experiments (Vaisman and Pelled, 2009), the authors argue that chronic administration of PS-DHA results in greater DHA accretion in the cortex compared with tri-DHA, indicating further improved DHA bioavailability. PS-DHA was supplemented until euthanization at term-equivalent age, i.e., 10 days after delivery. Supplementing preterm pigs with PS-DHA did not increase overall weight gain, but did significantly restore the decline in weight of the cerebellum at term-equivalent age up to the weight of term control pigs. PS-DHA supplementation also resulted in higher DTI indices in different brain regions, including the internal capsule, thalamus, hypothalamus, globus pallidus, hippocampus, and cortex. This indicates that a higher degree of myelination was present in brains of preterm pigs that were supplemented with PS-DHA as compared to placebo. Additionally, PS-DHA supplementation accelerated development of recognition memory and the auditory pathway as compared with placebo-treated preterm piglets.

In summary, these pre-clinical studies demonstrate that peripheral DHA supplementation effectively reduced lesion size and improved neurodevelopmental outcome in rodent models of neonatal HIE, when administered prior to of soon after the insult. In addition, DHA supplementation reduced lipid peroxidation markers in a piglet model of severe hypoxia-reoxygenation, indicating that DHA has anti-oxidative effects in the brain. In preterm-born piglets, enteral supplementation with DHA improved white matter integrity and cerebellar growth.

#### Clinical Trials

In total, five trials that described outcome after an intervention with lipids were included. Of those, one trial evaluated the effect of different amounts of lipids (Ong et al., 2018), three trials focused on supplementation with fatty acids (Almaas et al., 2015, 2016; Alshweki et al., 2015), and in one trial supplementation with sphingomyelin was used (Tanaka et al., 2013).

Ong et al. (2018) described the only included study in which different quantities of regular *parenteral* lipids were studied. They explored the impact of low (1 g/kg/day) and standard (3 g/kg/day) dose *parenteral* lipids (soybean oil, Intralipid) on growth and neurodevelopmental outcome until 2 years corrected age. No difference was found in neurodevelopmental outcome or growth up to 2 years of age between the groups (*n* = 30 in total) that received low or high dose soybean, with an exception for 12 months corrected age, at which a significant higher cognitive composite score was found in the patients with low dose soybean oil.

Supplementation with the fatty acids DHA and arachidonic acid (AA) was investigated in three included studies. In two papers, Almaas et al. (2015, 2016) described (1) cognitive (*n* = 98 in total) and brain volume outcome (Almaas et al., 2015) (*n* = 81 in total), and (2) Diffusion Tensor Imaging (*n* = 82 in total), and behavioral outcome (Almaas et al., 2016) (*n* = 98 in total) at 8 years of age in infants supplemented with enteral DHA and AA (ratio 1/1). Supplementation was initiated when the infant reached enteral feeding of 100 ml/kg/day and consisted of 0.5 ml study oil on 100 ml milk, which contained 32 mg DHA and 31 mg AA or placebo. Intervention ended when the patient was discharged or when a total of 100 ml of study oil was given. At 8 years of age outcome on brain volumes, cortical volumes, surface areas, thickness and white matter tract development as well as cognitive and motor outcome was similar between the fatty acid-supplemented and placebo patients. Alshweki et al. (2015) investigated the effect of AA/DHA ratio on outcome. They provided infants with enriched AA formula (AA/DHA ratio 2/1) or regular formula (ratio 1/1) and attempted to maintain this ratio in infant feeding during the entire follow up period of 2 years (*n* = 45 in total). A group of 25 infants that received exclusively breast milk were used as control group. At 2 years of age, the infants that received enriched AA formula had a higher score on the Brunet Lézine scale (Scale of Psychomotor Development of Children) compared to infants that received standard infant formula. Additionally, compared to breastfed controls, AA-enriched formula infants had similar Brunet Lézine scales whereas regular formula infants had significantly lower scores.

Tanaka et al. (2013) focused on supplementation with sphingomyelin. Infants (*n* = 24 in total) received either sphingomyelin-fortified enteral feeding (sphingomyelin 20% of all phospholipids in milk) or standard enteral feeding (sphingomyelin 13% of all phospholipids in milk). At 3–18 months of age infants in the intervention group scored better on the preference rate on the Fagan test and the sustained-attention test and showed shorter latency of visual evoked potentials. No differences in mental or psychomotor development were found.

In summary, in the one study that we included on quantities of lipids, no difference between high and low dose soybean oil was found at 2 years of age (*n* = 30 in total). DHA and AA supplementation showed no effect on brain volumes, brain maturation, and neurodevelopmental outcome (*n* = 98 in total). High AA/DHA ratio (2/1) did show a positive effect on Brunet Lézine scale compared to regular AA/DHA ratio (1/1) (*n* = 45 in total). Sphingomyelin supplementation improved some neurodevelopmental outcome measures, but showed no effect on mental or psychomotor development.

### Probiotic Supplementation

#### Pre-clinical Trials

Although no pre-clinical studies were found on the use of probiotics to improve neurodevelopmental outcome after neonatal brain injury, one group investigated the effects of butyrate (Jaworska et al., 2017; Ziemka-Nalecz et al., 2017), a short-chain fatty acid synthesized by bacteria in the colon by fermenting otherwise non-digestible fiber. Butyrate enters the bloodstream and subsequently the brain where it serves as a histone deacetylase (HDAC) inhibitor, activates G protein-coupled receptors, and acts as an energy metabolite to produce ATP. In two publications, the authors demonstrated in rats with neonatal HIE induced at P7 that i.p administration of 300 mg/kg sodium butyrate for five consecutive days post-HI reduced lesion size and neuroinflammation, as measured by levels of IL-1β and chemokine CXCL10 and polarization of microglia from pro-inflammatory M1 to anti-inflammatory M2 (Jaworska et al., 2017; Ziemka-Nalecz et al., 2017). In addition, butyrate treatment enhanced the generation of newly formed neuroblasts and oligodendrocytes in the dentate gyrus of the hippocampus of neonatal HIE rats, as compared to vehicle treatment. This neuroregenerative effect of butyrate is potentially driven by enhanced endogenous production of BDNF in the ipsilateral hemisphere. However, butyrate treatment failed to improve neurobehavioral outcome, as measured by behavioral tasks for motor function, spatial memory and social communication.

#### Clinical Trials

Three trials were included that used different (combinations of) bacteria as probiotic supplementation (Chou et al., 2010; Sari et al., 2012; Jacobs et al., 2017). Chou et al. (2010) supplemented preterm infants (*n* = 301 in total) with Infloran, containing *Lactobacillus acidophilus* and *Bifidobacterium infantis*, or placebo from 1 week after birth until discharge. Follow up at 3 years showed no difference in neurodevelopmental outcome between both groups. Jacobs et al. (2017) included and randomized patients (*n* = 735 in total) in receiving a combination of *Bifidobacterium infantis, Streptococcus thermophiles*, and *Bifidobacterium lactis* or placebo, from 72 h after birth until discharge or term equivalent age. Outcome was assessed between 2 and 5 years of age and no difference was found between both groups. Sari et al. (2012) evaluated the effect of supplementation of all feedings until discharge with *Lactobacillus Sporogenus* on neurodevelopmental outcome at 18–22 months corrected age (*n* = 174 in total). Again, no effect of supplementation on neurodevelopmental outcome was observed.

In total, all three studies combined evaluated 1,210 patients. In none of the studies, a significant effect of supplementation with probiotics on neurodevelopmental outcome was found.

### Prebiotic Supplementation

#### Pre-clinical Trials

No pre-clinical studies were found on prebiotic supplementation for the treatment of neonatal brain injury.

#### Clinical Trials

Two papers described neurodevelopmental outcome after supplementation with prebiotics (LeCouffe et al., 2014; van den Berg et al., 2016). Both papers assessed the same cohort, but at different time points. LeCouffe et al. (2014) evaluated outcome in the first year of life (*n* = 93 in total), whereas van den Berg et al. (2016) described outcome at 2 years corrected age (*n* = 77 in total). Intervention consisted of small-chain galacto-oligosaccharides (scGOS), long-chain fructo-oligosaccharides (lcFOS) and pectin-derived acidic oligosaccharides (pAOS) compared to controls from day 3 until day 30 post-natal. During the entire follow up period of 2 years, no significant difference was found in neurodevelopmental outcome between the patients with and without prebiotics supplementation.

### Vitamin Intake

#### Pre-clinical Trials

The effects of vitamins on neonatal brain injury were described in two pre-clinical studies (Miura et al., 2009; Ramani et al., 2017). The first study investigated whether a combination of vitamin A and its derivate retinoic acid attenuated hyperoxia-induced preterm brain injury in mice (Ramani et al., 2017). Chronic hyperoxia from P2–14 (equivalent to 24–40 weeks gestational age in humans) induced deficits in spatial memory and reductions in hippocampal volume and hippocampal synaptophysins. Enteral treatment with vitamin A (0.05 mmol/g) and retinoic acid (0.005 mmol/g) every other day during the hyperoxic period partially restored brain injury and behavioral outcome in mice subjected to hyperoxia. The authors suggested that neuroprotection by vitamin A plus retinoic acid was mediated via increased signaling of the mTOR pathway.

In the second study, the effect of vitamin C (i.e., ascorbic acid) was tested in a rat model of neonatal HIE induced at P7 (Miura et al., 2009). Treatment with 750 mg/kg of vitamin C i.p. prior to hypoxia reduced macroscopical brain injury and apoptotic and necrotic cell death in the cortex, hippocampus, caudate putamen, and thalamus of rats with neonatal HIE, as compared to vehicle. Microscopical analysis of lesion size was not examined in this study.

#### Clinical Trials

Only one RCT evaluated the effect of vitamin supplementation on neurodevelopmental outcome. Salas et al. (2018) studied the impact of two different doses of vitamin D (200 or 600 IU/day) compared to placebo from day 1 of enteral feeding until day 28 post-natal on neurodevelopmental outcome at 2 years of age (*n* = 91 in total). They found no significant difference in neurodevelopmental impairment (including cerebral palsy), cognitive impairment and language impairment between patients in the placebo group, the low dose supplementation group or the high dose supplementation group.

### Mineral Intake

#### Pre-clinical Trials

The effects of magnesium sulfate (MgSO_4_) supplementation were described in two pre-clinical studies from the same group (Koning et al., 2017; Seyama et al., 2018), using a rat model of neonatal HIE induced at P6 or P7. The first study in P7 rats demonstrated that the optimal dose of 1.1 mg/g MgSO_4_ i.p. effectively reduced lesion size when given as a pre-treatment between 6 days and 12 h prior to the insult, but not at later timepoints (Koning et al., 2017). In addition, mRNAs and miRNAs involved in metabolism and mitochondrial function were altered and mitochondrial respiration was preserved upon MgSO_4_ supplementation of neonatal HIE rats. MgSO_4_ pretreatment attenuated HI-induced increases in ROS production and neuroinflammation, as measured by cytokines and chemokine levels. The second study investigated MgSO_4_ pretreatment in a neonatal HIE rat model induced at P6 (equivalent to 29 weeks gestational age in humans) (Seyama et al., 2018). HI at this age generates selective decrease in myelination in the pericallosal white matter without overt gray matter loss, which more closely resembles non-cystic PVL. Pretreatment with MgSO4, 30 min before the hypoxic insult, attenuated hypomyelination of the ipsilateral pericallosal white matter, as compared to vehicle pre-treatment. This increase in myelination was associated with increased oligodendrocyte numbers *in vivo* and reduced pre-oligodendrocyte death *in vitro*. Therefore, the authors suggested that MgSO4 ameliorated the white matter damage by preventing cell death of pre-oligodendrocytes.

#### Clinical Trials

Williams et al. (2017) investigated neurodevelopmental outcome after iodide supplementation. Preterm infants (*n* = 1259 in total) were randomly assigned to receive either placebo (30 μg/kg/day sodium chloride) or iodide supplementation (30 μg/kg/day sodium iodide) from <42 h after birth until 34 weeks gestational age. Neurodevelopmental outcome at 2 years of age was not significantly different in the groups.

## Discussion

With this review we provided an overview of the currently available evidence for the effect of nutritional interventions on neurodevelopmental outcome from clinical RCTs in preterm infants and pre-clinical models of neonatal brain injury. Some clinical nutritional intervention studies showed beneficial short term effects, such as increased head circumference growth, but overall, there is little clinical evidence for long term positive effects of nutritional supplementation on neurodevelopmental outcome in preterm infants. Some clinical studies even showed adverse short term effects of increased nutritional intake. On the contrary, all the included pre-clinical studies found positive effects of nutritional supplementation on brain injury and/or neurodevelopment.

The most promising clinical results were seen in RCTs focusing on supplementation with glutamine, that showed improved head circumference growth during the first year of life and increased brain volumes at 8 years of age. However, at 13 years of age neurodevelopmental outcome was similar between glutamine-supplemented infants and controls. Early and/or high parenteral amino acid supplementation resulted in inconsistent outcome, with no effect on neurodevelopment, but both neutral and negative effects on growth and head circumference. Enteral protein supplementation increased head circumference growth at term equivalent age, but showed no effect on neurodevelopment. Overall lipid intake or increased DHA and AA intake (ratio 1/1) did not improve neurodevelopmental or brain injury outcome, but increased AA/DHA ratio (2/1) normalized neurodevelopmental outcome to the level of breast fed term controls. No positive or negative effect of supplementation with probiotics, prebiotics, vitamin D, and iodide were found in clinical trials. In pre-clinical models, less brain injury and/or improved behavioral outcome was seen after supplementation with lactoferrin, acetyl-l-carnitine, taurine, glycine, l-cysteine, DHA, butyrate, vitamins A and C, and magnesium sulfate.

The clinical part of our systematic review is in line with findings from previously published systematic reviews or meta-analyses regarding neurodevelopment following nutritional intervention. Both Chan et al. (2016) and Schneider and Garcia-Rodenas (2017) included studies with heterogeneous interventions in their systematic reviews and/or meta-analysis that showed conflicting results with regard to (individual) nutritional components (Chan et al., 2016; Schneider and Garcia-Rodenas, 2017). To our knowledge, this is the first systematic review of nutritional interventions in pre-clinical models of neonatal brain injury.

Also in term infants supplementation with different nutritional components, such as probiotics, prebiotics and fatty acids, has been tested. Bertelsen et al. reviewed the evidence regarding probiotic and prebiotic supplementation in relation to development of the gut microbiome (Bertelsen et al., 2016). In that review, brain injury or neurodevelopment was not assessed. A systematic Cochrane review by Jasani et al. summarized the evidence of supplementation with fatty acids and their effect on neurodevelopment (among other things) (Jasani et al., 2017). The authors conclude that there is no beneficial effect or harm of supplementation of fatty acids in relation to neurodevelopmental outcome. Another interesting study population that this review did not specifically focus on is the intra-uterine growth restricted (IUGR) infant. These infants are, for different reasons, already *in utero* deprived of a sufficient amount of nutritional components to thrive adequately. Therefore, IUGR infants might benefit extra from nutritional interventions post-partum. Since the population of IUGR infants is heterogeneous in GA, it was beyond the scope of this review to include studies on term IUGR infants specifically. However, since we selected RCTs based on GA or birth weight, IUGR preterm infants were included in our search. A systematic review specifically focused on nutritional interventions in IUGR infants would be of great relevance to address brain injury in this population.

An interesting observation in this review is the difference between clinical and pre-clinical study outcomes. All pre-clinical studies described positive outcomes after nutritional supplementation, while almost no clinical studies found a positive result. This could be due to several factors. First of all, long term neurodevelopmental outcome in clinical studies is much more dependent on environmental factors compared to a pre-clinical study, in which the environment of the animals is strictly regulated. Long term follow up in preterm infants is performed one or more years after the intervention. In this time frame, environmental factors such as socioeconomic status, which is an important predictor for later neurodevelopment (Linsell et al., 2015), and post-discharge nutritional intake, cannot (or hardly) be regulated. It is therefore more difficult to detect an effect of a clinical intervention on long term outcome, especially when the intervention is given for a short period of time (usually during a few days or weeks after birth). Secondly, similar to the heterogenic environment, the population of preterm infants is much more heterogenic than a controlled animal population. Preterm infants could be suffering from all kinds of short and long term complications, such as sepsis, intraventricular hemorrhage, pulmonary or gut problems or they can have a relatively uneventful course. These complications can have a major impact on later development. For instance, bronchopulmonary dysplasia is an important risk factor for adverse neurodevelopment and academic performance (Twilhaar et al., 2018b,c). Furthermore, preterm infants can have different types of WMI, such as diffuse, cystic or hemorrhagic injury, which can in turn affect neurodevelopment differently. Additionally, nutritional intake of preterm infants can also be heterogenic, even in a controlled clinical trial. Preterm infants are often fed breast milk, but the content of preterm breast milk varies substantially, depending for example on maternal diet or post-natal day (Boyce et al., 2016; Mimouni et al., 2017). The quantity of nutrients in breast milk is often not measured nor taken into account in clinical nutritional intervention trials, which may lead to a clinically relevant difference in intake, effecting study outcome. Heterogeneity might be an important factor when interpreting the results of clinical studies and could also (partly) explain why there is a difference between clinical and pre-clinical results.

Thirdly, the main outcome focus of pre-clinical studies was brain injury. This is in contrast with clinical studies, in which neurodevelopment was the main outcome measure and only a few studies focused on brain injury parameters (for example brain volumes or white matter tract development). It is possible that clinical interventions demonstrate the same effects on brain development as pre-clinical interventions, but that this does not translate into improved neurodevelopmental outcome. Furthermore, most pre-clinical studies were conducted in models for neonatal HIE as observed in the term born infant. The timing of the insults is crucial for type of neurological outcome. Most of the included studies in this review subjected rodents to hypoxic-ischemic brain injury at P7. At this moment in rodent brain development, the white matter is in a state of maturation similar to human white matter between 30 and 36 weeks GA (Semple et al., 2013; Salmaso et al., 2014), meaning that oligodendrocytes have mostly matured and the stage at which EoP can be induced has already passed. Therefore, hypoxia-ischemia at P7 induces mainly neuronal death, as seen in perinatal asphyxia and neonatal stroke, but not an arrest of oligodendrocyte maturation, as is now commonly thought to be the major contributor to the development of EoP. In addition to timing, also inclusion of an inflammatory component is thought to be key to the development of translational animal models of EoP. In recent years, rodent models of EoP have been developed (Zeng et al., 2018), including several multiple hit models that combined fetal inflammation with post-natal hypoxia (van Tilborg et al., 2018a) or multiple post-natal injections of cytokines (Rangon et al., 2018). Also larger animal models of EoP have been developed, mostly using preterm lambs (Li et al., 2016; Wassink et al., 2017; Gussenhoven et al., 2018), piglets (Andersen et al., 2016; Buddington et al., 2018), and baboons (Griffith et al., 2012). Apparently, nutritional intervention studies have so far been scarcely conducted in pre-clinical models for EoP, as we included only four studies in rodent models of preterm EoP (van de Looij et al., 2014; Ginet et al., 2016; Ramani et al., 2017; Seyama et al., 2018) and one study in late preterm piglets (Buddington et al., 2018). Therefore, to move forward with the development of nutritional therapies for preterm infants, there is an urgent need for the use of these clinically relevant pre-clinical EoP models, which can hopefully improve the implementation of pre-clinical nutritional therapies in clinical practice in future.

From a statistical point of view, the absence of beneficial effects in clinical studies could also be due to the fact that almost none of the RCTs was powered to detect differences in neurodevelopmental outcome or brain injury. In these studies, long term effect of supplementation was a secondary or explorative outcome. It is therefore possible that effects of supplementation were present, but could not be detected. This is in contrast with the pre-clinical studies, in which brain injury and/or development was the primary study outcome. Furthermore, it is possible that the lack of neutral or negative results in the pre-clinical studies is due to publication bias.

Finally, the sensitivity of the clinical outcome measures could also be an issue in failure to detect positive effects. Because of their risk of adverse neurodevelopmental outcome, preterm infants are usually regularly seen for follow up in the clinic. One of the main issues in interpretation of the results of this systematic review is the value of this neurodevelopmental follow up. It remains difficult to predict neurodevelopmental outcome in extremely preterm children, even with the use of standardized outcome scales. The most commonly used outcome scale nowadays is the Bayley Scales of Infant and Toddler Development (BSITD), which has a first, second and third edition and consists, among other things, of a mental and motor scale. The BSITD is used to predict development at a later stage of life. However, a systematic review and meta-analysis by Luttikhuizen dos Santos et al. (2013) showed that the predictive value of the BSITD is limited (Luttikhuizen dos Santos et al., 2013). The mental score explains 37% of later cognitive functioning, while the motor score predicts only 12% of later motor functioning. Even though these percentages are low, they are used in clinical practice as well as in evaluating outcome of clinical trials. To get real insight into the effects of an early intervention on later functioning, longer follow up, perhaps even into adulthood, is needed. Furthermore, the goal of follow up is to monitor development and intervene when necessary. When delay in development is present or imminent, action is taken, such as physiotherapy or school support, to optimize the infant's change of normal development. This can influence the results of clinical trials evaluating outcome. This is in contrast with pre-clinical studies, when the natural course of outcome of the animals is followed, which will give a more reliable understanding of the effect of the intervention.

Our systematic review has some limitations. First of all, only studies that were published no longer than 10 years ago at the time of search were included and therefore older studies providing evidence regarding nutritional interventions have been missed. Considering the change in clinical pathophysiology of preterm brain injury from cystic periventricular leukomalacia to more diffuse forms of WMI in the past decades and the continuously improving clinical care, we focused on studies of the last 10 years, to have a relevant study population for the currently observed pathophysiology of EoP in preterm infants. Secondly, our review focused on brain injury and neurodevelopmental outcome after a nutritional intervention, but in some studies body and/or head circumference growth were also mentioned as an outcome measure. Especially head circumference growth is associated with neurodevelopmental outcome (Peterson et al., 2006; Franz et al., 2009). We therefore also mention the results of body and head circumference growth in this review. However, we have to take into account that we did not use growth or head circumference in the search strategy. Studies that solely focused on growth and not mentioned neurodevelopmental outcome can be missed with our search and the results on growth should therefore be interpreted with caution. Thirdly, as nutritional intervention studies have hardly been conducted in models for EoP, we included pre-clinical intervention studies done in all models for neonatal brain injury induced by hypoxia, ischemia, hyperoxia, and/or inflammation. Neonatal HIE is primarily characterized by gray matter injury, while in most types of EoP the white matter is mainly affected. This is an important difference in pathology that should be taken into account when testing nutritional interventions. Although the models also have similarities in pathophysiology on which nutrition has been shown to have effects, such as neuroinflammation and axonal injury, the interpretation of the pre-clinical data observed in neonatal models for HIE should be interpreted with caution. Finally, only studies that evaluated a single nutritional component were included. We thereby excluded multiple studies that looked at combined interventions and that could have given meaningful results. We chose this approach to be able to interpret our results directly related to the single nutrient that was supplemented.

## Conclusion

Even though many pre-clinical trials show beneficial effects of different nutritional interventions on brain injury and/or neurodevelopmental outcome, these positive effects have so far not clearly been demonstrated in RCTs. To move novel nutritional therapies for encephalopathy of prematurity from the bench to the bedside of preterm infants, there is an urgent need to investigate nutritional therapies for preterm infants in more translational and clinically relevant animal models. This review emphasizes the need for more consistent long term follow-up of preterm infants included in clinical trials and the development of better methods to evaluate neurodevelopmental outcome of this vulnerable population.

## Author Contributions

LH and CdT performed the searches, screened and selected papers, drafted the initial manuscript, and revised the manuscript. RvE supervised article selection and reviewed the manuscript critically for intellectual content. CN and MB critically reviewed the manuscript for intellectual content. All authors approved the final version of the manuscript and take full responsibility for the content.

### Conflict of Interest Statement

RvE is employed by Danone, Nutricia Research. The remaining authors declare that the research was conducted in the absence of any commercial or financial relationships that could be construed as a potential conflict of interest.

## Abbreviations

AA: arachidonic acid
BSITD: Bayley Scales of Infant and Toddler Development
DHA: docosahexaenoic acid
EoP: Encephalopathy of Prematurity
EPA: eicosapentaenoic acid
FA: fractional anisotropy
HIE: hypoxic-ischemic encephalopathy
HMF: human milk fortifier
PLIC: posterior limb of the internal capsule
RCT: randomized controlled trial
WMI: white matter injury.
